# Outcome of trauma-related emergency laparotomies, in an era of far-reaching specialization

**DOI:** 10.1186/s13017-019-0257-y

**Published:** 2019-08-14

**Authors:** Falco Hietbrink, Diederik Smeeing, Steffi Karhof, Henk Formijne Jonkers, Marijn Houwert, Karlijn van Wessem, Rogier Simmermacher, Geertje Govaert, Miriam de Jong, Ivar de Bruin, Luke Leenen

**Affiliations:** Department of Surgery, University Medical Center Utrecht, PO Box 85500, 3508 GA Utrecht, The Netherlands

**Keywords:** Trauma, Abdominal injury, Laparotomy, Complications, Survival, Retrospective cohort

## Abstract

**Background:**

Far reaching sub-specialization tends to become obligatory for surgeons in most Western countries. It is suggested that exposure of surgeons to emergency laparotomy after trauma is ever declining. Therefore, it can be questioned whether a generalist (i.e., general surgery) with additional differentiation such as the trauma surgeon, will still be needed and can remain sufficiently qualified. This study aimed to evaluate volume trends and outcomes of emergency laparotomies in trauma.

**Methods:**

A retrospective cohort study was performed in the University Medical Center Utrecht between January 2008 and January 2018, in which all patients who underwent an emergency laparotomy for trauma were included. Collected data were demographics, trauma-related characteristics, and number of (planned and unplanned) laparotomies with their indications. Primary outcome was in-hospital mortality; secondary outcomes were complications, length of ICU, and overall hospital stay.

**Results:**

A total of 268 index emergency laparotomies were evaluated. Total number of patients who presented with an abdominal AIS > 2 remained constant over the past 10 years, as did the percentage of patients that required an emergency laparotomy. Most were polytrauma patients with a mean ISS = 27.5 (SD ± 14.9). The most frequent indication for laparotomy was hemodynamic instability or ongoing blood loss (44%).Unplanned relaparotomies occurred in 21% of the patients, mostly due to relapse of bleeding. Other complications were anastomotic leakage (8.6%), intestinal leakage after bowel contusion (4%). In addition, an incisional hernia was found in 6.3%. Mortality rate was 16.7%, mostly due to neurologic origin (42%). Average length of stay was 16 days with an ICU stay of 5 days.

**Conclusion:**

This study shows a persistent number of patients requiring emergency laparotomy after (blunt) abdominal trauma over 10 years in a European trauma center. When performed by a dedicated trauma team, this results in acceptable mortality and complication rates in this severely injured population.

## Background

In the past decades, it is suggested that the exposure of most European surgeons to an emergency laparotomy in severely injured patients is declining [1]. Penetrating injuries are a frequent indication for laparotomy in trauma patients [2, 3]. However, in most European countries, penetrating injuries are a rare phenomenon. In addition, most blunt trauma patients can nowadays be managed by non-operative management (NOM) [1, 4, 5]. This is in contrast to countries such as the USA and South Africa where exposure is higher due to high-volume presentation of trauma patients with penetrating abdominal injuries [1, 3, 6, 7]. The current challenge is to provide an adequate level of care with satisfactory outcome for trauma patients who require an emergency laparotomy in Europe [8].

In an era of ongoing sub-specialization, trauma surgery still requires a broad perspective and diverse skill set in order to provide adequate care for the most severely injured patients [5]. However, nowadays, surgeons tend to specialize more than ever before into small areas of interest, something which is encouraged by governments and insurance companies. Furthermore, the reluctance of far-reaching centralization in trauma in combination with work hour regulations restrict exposure for surgeons in the European Union for this procedure [9]. Therefore, it can be questioned if the knowledge and skillset required to perform such an emergency laparotomy in severely injured patients can be maintained [10]. These developments might challenge the competence of the surgeons who deal with trauma patients with regard to an emergency laparotomy and, as a result, outcome of severely injured patients [11].

A major asset in the treatment of a trauma patient is knowledge of damage control concepts. Damage control surgery (DCS) can be performed in severely injured patients as part of the resuscitation process [12]. In DCS, the goal is to reduce operating time as much as possible, preferably within 1–1.5 h, in which hemorrhage and contamination is controlled, while additional damage is prevented [13]. Thereby limiting the lethal triad in trauma consisting of coagulopathy, hypothermia, and acidosis and provide the possibility to restore physiology [13]. A single procedure is often not sufficient to gain control and patients are frequently brought back to the operating room for relaparotomy in support of further resuscitation. Although the damage control concept has reduced mortality over the last decades [14, 15], high morbidity and mortality rates remain, especially in patients who arrive in extremis at the emergency department [2, 3, 16]. The mortality rate of patients undergoing a trauma laparotomy is reported up to 21%, with exsanguination accounting for 60% of these deaths, even in high volume centers [2].

The aim of this study was to evaluate the outcome after an emergency laparotomy in trauma patients, performed in a Dutch level 1 trauma center.

## Methods

A published study protocol does not exist. A single-center retrospective observational cohort study was performed in the University Medical Center Utrecht (UMCU), a large teaching hospital and designated level I trauma center in the Netherlands.

In our hospital, the team of trauma surgeons exists of 5–8 trauma surgeons (depending on the time period within the study) with one or two trauma fellows. In the past years, a trauma surgeon is physically present in the hospital 24 h, 7 days a week. In our center, all crash-room activated trauma calls and subsequent surgical procedures (both truncal and musculoskeletal) are directly supervised or carried out by a certified trauma surgeon, with a background in general surgery [17]. We have approximately 1800 trauma activations per year with nearby 200 per year arriving in severe shock. However, this depends on the definition of ‘in shock.’ A bit more patients are triaged in ‘Red’ (300 per year), compared to being in shock (220) based on the clinical parameters in the hospital (after resuscitation by the paramedics and Mobile Medical Team by helicopter). Of all trauma team activations, 1400 patients are admitted yearly with on average 365 of them for polytrauma (ISS > 15), above that we admit 400 complex mono injuries (mostly after high energy mechanism) and 500 isolated neurologic injuries every year. We have a strong lateralization in our inclusive trauma system, with most non-complex injuries being treated in the level 2 and 3 hospitals in the region.

Every laparotomy following trauma is performed by two trauma surgeons. All together, this means a trauma surgeon performs an average of five to ten index laparotomies for trauma per year in our center. Additional procedures after the index operation come on top of this including delayed reconstructions. To maintain the skill set needed for a trauma surgeon, every member of the staff is current at Definitive Surgical Trauma Care Course (DSTC)® and several are instructors on this course. Furthermore, we perform additional annual training in the wet lab and two times per year we do cadaver training with our team.

### The performance of a laparotomy in trauma and damage control surgery

Damage control surgery in trauma patients has been previously extensively described [18, 19] as has the concept of Dutch trauma surgery [20]. The indication for an emergency laparotomy is based on patient’s physiology as indicated by vital signs (pulse rate, blood pressure, urine output, temperature) and laboratory findings (coagulation, base deficit, hemoglobin levels) [21]. The objective is to complete the procedure within 60–90 min [17]. If appropriate, the abdomen is temporary closed, preferably with a vac-pack [22]. Furthermore, the trauma surgeon is actively involved in the subsequent ongoing resuscitation of trauma patients in intensive care unit (ICU) and in the indication and timing of further surgical procedures, either truncal or skeletal [23]. The re-laparotomy for definitive surgical care, if indicated, is planned as soon as physiologic (mainly hemodynamic) stability was achieved. An unplanned re-laparotomy was defined as a laparotomy forced by deterioration of the patient, such as ongoing or renewed blood loss, intra-abdominal infection, bile leakage, or fascial dehiscence.

A systemic approach was used to perform the initial emergency trauma laparotomy in hemodynamically unstable patients as described previously. In short, laparotomy is started with midline incision from xiphoid process to the pubic bone. After opening the abdominal cavity, blood and blood clots are removed. All quadrants of the abdomen are then systematically packed using large gauzes. Each abdominal quadrant is inspected for injuries. Surgical management is performed to stop the bleeding and to prevent (further) contamination of the abdomen. If arterial bleeding is insufficiently controlled, additional angioembolization is utilized. Unfortunately, no hybrid operation room (OR) is structurally available for trauma in our hospital, resulting in extra transport times for (combined) endovascular procedures.

### Patient selection

All consecutive patients who underwent an emergency laparotomy directly related to trauma between January 2008 and January 2018 were included. All patients were initially assessed and treated following Advanced Trauma Life Support (ATLS) guidelines by dedicated Dutch trauma surgeons in a Level-1 trauma center (trained in both general surgery and orthopedic surgery for trauma). Patients were identified using the hospital surgical registration system. Patients of all ages were included. All data were extracted from the prospective trauma center database and supplemented with information from the electronic patient medical records.

### Study variables

The collected data of the included patients were demographics: age, gender, weight, length, body mass index (BMI), American Association of Anesthesiologists (ASA) classification, smoking, diabetes, previous abdominal surgery, and cardiopulmonary history. If comorbidities were not noted in the electronic patient file, it was assumed as being absent in the patient. This means that for instance diabetes was assumed absent if the patient was not under supervision of a doctor for treatment, and did not use any medication for diabetes nor diabetes was mentioned in the medical history. ASA classification was calculated based on the known comorbidities at time of trauma. Furthermore, the following trauma-related characteristics were noted: the condition of the patient at arrival and their injuries were noted, trauma mechanism (fall lower than 3 m, fall higher than 3 m, car accident, motorcycle accident, scooter accident, bicycle accident, pedestrian accident, stabbing, shooting, entrapment, and other causes), alcohol consumption at the moment of the event, Injury Severity Score (ISS), relevant blood results (hemoglobin, pH, and base deficit), Glasgow Coma Scale (GCS), and systolic blood pressure, the latter two both on arrival in ED. The ISS was calculated through trauma registry specialists and verified by dedicated trauma staff. In addition, the number of (planned and unplanned) laparotomies per patient were collected and the indication for any surgical intervention was noted. Other collected data were presence of a CT-scan prior to laparotomy, time to laparotomy, found injuries, procedure time, and procedures performed. If the inferior caval vein, aorta, celiac artery, superior mesenteric artery, superior mesenteric vein, or iliac veins were involved, it was noted as large vessel injuries. An unplanned re-laparotomy was defined as any laparotomy performed after the initial trauma laparotomy but not planned to provide (further) definitive surgical care. All variables were established before data extraction was performed.

### Outcome variables

The in-hospital mortality was noted as primary outcome. In addition, the cause of mortality was extracted from the records. Secondary outcomes were complications, length of ICU, and overall hospital stay. Complications included unplanned re-laparotomies, re-bleeding, and failure of intestinal anastomosis.

### Data and statistical analysis

Continuous parametric data were presented as means with standard deviations; continuous non-parametric data were presented as medians with interquartile ranges (IQRs); dichotomous and categorical data as frequencies with percentages. The cause of in-hospital mortality was stratified by GCS and the systolic blood pressure on arrival. A *P* value < 0.05 was considered statistically significant. All statistical analyses were performed using Statistical Package for the Social Sciences (IBM Corp. Released 2011. IBM SPSS Statistics for Windows, Version 20.0. Armonk, NY: IBM Corp.). Data analysis was performed with full anonymity of the included patients. This study was performed according to the Institutional Review Board (IRB) medical ethics standards. The IRB concluded that approval by an ethics committee was not applicable and a waiver was provided (16-702/C).

## Results

### Demographic data

**Fig. 1:**
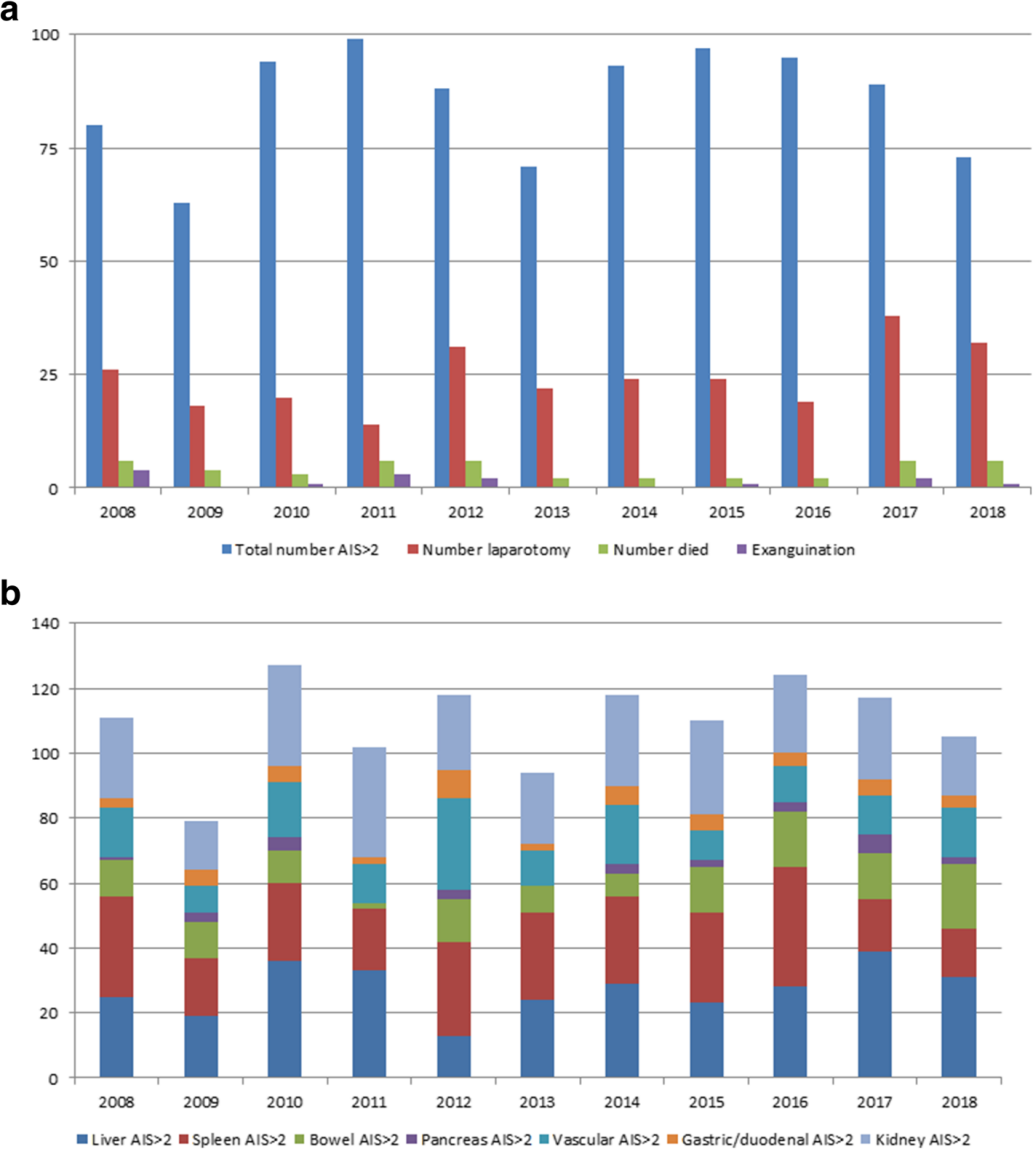
Number of patients and mortality rates per studied year. This figure shows **a** all patients with an abdominal AIS above 2 (blue bars), the number of injured patients that required an emergency laparotomy (red bars), the number of patients with abdominal trauma that died (green bars), and the number of patients with abdominal trauma that died due to exsanguination (purple bars). These results are depicted per studied year, which shows that the total number of abdominal injuries dictating laparotomy remained similar over the years, as well as the differentiation of the individual injuries found (**b**). Furthermore, the mortality due to exsanguination was further minimalized over the past years

During the study period, 267 consecutive trauma patients underwent 268 index emergency laparotomies directly after or as part of resuscitation. The mean age was 42.5 years (± 30.8, range 3 to 83 years). Twenty patients were younger than 18 years. Most patients were males (*n* = 177; 66%). Twenty-three percent (*n* = 63) of the studied population suffered from psychiatric illness that required psychiatric support. Furthermore, most patients could be considered as healthy with an ASA 1 or 2 classification and a limited number of comorbidities (Table 1). The total number of patients who presented with an abdominal AIS > 2 remained similar over the past 10 years (Fig. 1a, b) as did the percentage of patients who required an emergency laparotomy.

**Table 1:** Baseline characteristics patients

	Total (*n* = 268)	Survived (*n* = 223)	Deceased (*n* = 45)	*P* value
Age in years, mean (± SD)	42.5 (± 30.8)	40.2 (± 17.8)	50.8 (± 24.2)	0.001
Male/female	177/91	152/71	24/21	0.046
ASA classification (missing 9)				0.081
- ASA 1	139	124	15	
- ASA 2	83	66	17	
- ASA 3	32	28	4	
- ASA 4	5	3	2	
Psychiatric comorbidities (missing 16)	63	55	8	NS
History of abdominal surgery (missing 10)	18	16	2	NS
History of diabetes (missing 10)	17	12	5	NS
History of cardiac disease (missing 10)	36	27	9	0.054
History of pulmonary disease (missing 10)	26	20	6	NS
Trauma mechanism (missing = 0)				NS
- Fall < 3 m	14	13	1	
- Fall > 3 m	22	18	4	
- MVA car	74	62	12	
- MVA motorcycle	15	13	2	
- MVA bike	59	45	14	
- MVA pedestrian	8	6	2	
- Penetrating trauma	49	43	6	
- Gunshot wound	9	9	0	
- Crush injury	7	5	2	
- Other	11	9	2	
Suicide attempt	44	38	6	NS
ISS, mean (± SD)	27.5 (± 14.9)	25.2 (± 13.8)	38.7 (± 15.8)	<0.001
AIS regions				
- AIS head	1.07 (± 1.6)	0.9 (± 1.5)	1.8 (± 2.0)	0.006
- AIS face	0.27 (± 0.7)	0.3 (± 0.7)	0.3 (± 0.8)	NS
- AIS chest	2.24 (± 1.8)	2.1 (± 1.8)	3.1 (± 1.6)	0.001
- AIS abdomen	2.74 (± 1.5)	2.7 (± 1.5)	3.0 (± 1.8)	NS
- AIS extremities	1.55 (± 1.6)	1.4 (± 1.6)	2.1 (± 1.6)	0.015
- AIS external	0.81 (± 0.8)	0.8 (± 0.8)	0.9 (± 1.0)	NS
Glasgow Coma Scale, mean (± SD)	10.5 (± 5.3)	12.5 (± 4.1)	4.5 (± 3.6)	<0.001
Systolic blood pressure (mmHg)	108 (± 33)	114 (± 27)	77 (± 40)	<0.001
Hemoglobin (mmol/ml)	7.28 (± 1.9)	7.7 (± 1.7)	5.1 (± 1.8)	<0.001
pH	7.26 (± 0.17)	7.32 (± 0.12)	7.05 (± 0.19)	<0.001
Base deficit	5.8 (± 7.6)	3.7 (± 5.9)	15.4 (± 7.3)	<0.001

Baseline characteristics of all patients that have underwent an emergency laparotomy (*N*=268) divided in patients who survived (*N*=223) and patients that deceased (*N*=45). Significant differences are mean age, gender, and severely injured patients as shown by higher ISS and AIS and vital parameters at presentation. NS = not statistically significant (p>0.10)

### Injury mechanism and severity

The main causes of trauma were road traffic accidents (*n* = 156; 58%) followed by penetrating injuries (*n* = 58; 21%) and falls (*n* = 36; 13%). Most patients were polytrauma patients, who also suffered head, chest, pelvic, and/or extremity injuries (Table 1). The mean ISS was 27.5 (± 14.9; range 1 to 75). This also resulted in a severe physiological derangement in most patients, expressed by a mean systolic blood pressure of 108 mmHg (± 33), a GCS of 10 (± 5), a hemoglobin of 7 (± 2) mmol/L, a pH of 7.26 (± 0.17), and a base deficit of 5.8 (± 7.6) mEq/L.

### Injuries and treatment

The most frequent indication for laparotomy was hemodynamic instability or ongoing blood loss from either an intra-abdominal or retroperitoneal source during resuscitation. Nearly all patients were in the operating room within 2 h, while 62% of the patients underwent pre-operative CT-scanning (Table 2). The 38% without CT-scanning prior to surgery were prompted for laparotomy either by hemodynamic instability (*n* = 99) or penetrating injury (*n* = 26). A wide variety of injuries was found, with spleen, liver, and colon injuries in at least a quarter of all patients. Furthermore, retroperitoneal vascular injuries occurred frequently and an assessment was made to resect or wait-and-see on bowel or mesenteric contusion in 94 patients (35%). Damage control procedures with abbreviated surgery were performed in 105 patients (39%). The average time of a laparotomy was 62 min (± 30). One hundred nine patients (41%) had two or more laparotomies, with a total number of 484 abdominal explorations performed. Surgical procedures during these explorations were diverse, including packing, intestinal resections, vascular repair, and intestinal anastomosis. Surgical treatment of a delayed cecum blow-out occurred in seven patients (2.6%). Intestinal anastomosis was performed in 46 patients with a total of 62 anastomoses. Forty laparotomies were considered non-therapeutic, which were divided on both penetrating and blunt trauma patients. Sixteen of these non-therapeutic laparotomies were for penetrating injuries. Another six patients underwent a laparotomy for severe abdominal pain, which afterwards appeared negative. The remaining negative laparotomies were performed in patients in extremis, following a resuscitation thoracotomy or an inconclusive FAST. There was no significant relationship between negative laparotomy and the usage of pre-operative CT-scan in penetrating trauma, while in the latter patient category, no CT-scan was performed due to the physiological condition of the patient not allowing us to do so.

**Table 2:** Baseline characteristics laparotomy

	Total (*n* = 268)	Survived (*n* = 223)	Deceased (*n* = 45)	*P* value
Indication for laparotomy				
- Hemodynamic instability	107	68	39	<0.001
- Penetrating injury*	42	41	2	
- Suspicion of hollow viscus injury	61	59	2	
- Ongoing blood loss	24	21	3	
- Retroperitoneal (pancreas/duodenum/vascular)	11	11	0	
- Severe abdominal pain	10	10	0	
- Suspicion of diaphragm injury	5	5	0	
- Ileus after trauma	6	6	0	
- Abdominal compartment syndrome	2	2	0	
CT-scan before laparotomy (missing = 2)	168	154 (69%)	14 (31%)	<0.001
Average time to laparotomy > 24 h (days)		5.6 (± 4.8)	7.7 (± 7.6)	NS
Time to laparotomy (hours)				
0–2 h	190	149	41	
2–6 h	17	16	1	
6–24 h	24	24	0	
> 24 h	37	34	3	
Injuries found (numbers)				
- Spleen	87	71	16	NS
- Liver	71	56	15	NS
- Stomach	19	13	6	0.073
- Pancreas	12	10	2	NS
- Duodenum	11	9	2	NS
- Colon defect	70	62	8	0.091
- Short bowel defect	52	47	5	0.084
- Bowel/mesentery contusion	94	77	17	NS
- Kidney	30	25	5	NS
- Bladder	8	5	3	NS
- Diaphragm	28	20	8	0.068
- Vascular injury	47	29	18	< 0.001
- Retroperitoneal major vascular	63	44	19	0.002
- Abdominal wall defect	28	24	4	NS
- Pelvic fractures	54	43	11	NS
- Secondary caecum blowout	7	6	1	NS
Time of procedure (min)	62.0 (± 30.0)	61.5 (± 28.8)	61.4 (± 32.5)	NS
Number of laparotomies per patient	1.8 (± 1.4)	1.8 (± 1.5)	1.5 (± 0.8)	
Procedures during laparotomy (*n*)	486			
- Damage control surgery	105	67	38	<0.001
- Non-therapeutic laparotomy	40	35	5	NS
- Abdominal packing	111	77	34	<0.001
- Splenectomy/Spleen preserving	53	41	12	NS
- (Partial) hepatectomy	2	2	0	NS
- Liver repair (sealants, sutures)	17	15	2	NS
- Nephrectomy/renal repair	7	4	3	NS
- Gastric repair	16	12	4	NS
- Colonic resection	35	29	6	NS
- Colonic repair (suture)	48	46	4	0.049
- Short bowel resection	26	22	4	NS
- Short bowel repair (suture)	36	32	4	NS
- Anastomosis (colon/short bowel)	46	44	4	0.035
- (Partial) pancreatectomy	2	1	1	NS
- Duodenal repair (anastomosis)	5	4	1	NS
- Pancreatic/duodenal suture/drainage	10	9	1	NS
- Urinary bladder repair	6	5	1	NS
- Vascular ligation (small vessels)	55	41	14	0.041
- Vascular repair (large vessels)	13	4	9	<0.001
- Diaphragm repair	29	21	8	0.082
- Evacuation bile/hematoma	4	4	0	NS
- Abdominal wall repair	20	19	1	NS
LOS intensive care (days)	5.12 (± 8.8)	6.06 (± 9.9)	3.39 (± 4.9)	0.002
LOS Hospital (days)	16.2 (± 20.6)	19.4 (± 21.7)	3.72 (± 5.3)	<0.001

Baseline characteristics of all emergency laparotomies performed (*N* = 268), comparing characteristics of the ones who survived (*N* = 223) to those that did not (*N* = 45). Significant parameters were indication for laparotomy with hemodynamic instability having the highest number of deaths, CT scan before laparotomy, (retroperitoneal) vascular injuries, and some of the procedures performed were seen more in the deceased population. Length of stay in hospital or ICU was significantly longer for the patients who survived

### Outcome analysis

The average length of stay was 16 days (SD 20.4), with an ICU stay of 5 days (SD 8.8). A total of 45 (16.7%) patients deceased during their hospital stay. Most patients deceased due to central nervous system failure after severe brain injury (19 out of 45 deaths; 42%, Tables 3 and 4). Patients who arrived with a systolic blood pressure below 90 mmHg had a higher mortality rate (26 out of 69; 37%) compared to patients with a systolic pressure of 90 mmHg or higher (19 out of 199; 9%, *P* < 0.001). Patients who died were older, had a higher ISS, and worse physiology (by GCS, systolic blood pressure, hemoglobin, pH, base deficit) compared to those who survived (Table 2). The other main cause of death was persistent blood loss, either retroperitoneal in diffuse injuries, or more specifically from aorta or (retro-hepatic) caval vein injuries (Table 2).

**Table 3:** Mortality by Glasgow Coma Scale

	Glasgow Coma Scale		
	<9	9–14	15
Total number of patients^a^	*n* = 72	*n* = 56	*n* = 134
Total number of deaths^b^	38	3	4
Cause of mortality			
Central nervous system	17	1	1
Exsanguination	5	2	0
Retroperitoneal exsanguination	6	0	1
Multi organ failure	3	0	1
Chest injury	3	0	0
Cardiac failure	1	0	0
Respiratory insufficiency	0	0	1
Pre-existing condition	2	0	0
Other	1	0	0

Cause of in-hospital mortality by Glasgow Coma Scale (GCS)

^a^Total *N* = 262, missing six patients in whom GCS has not been documented

^b^Of all patients that died, GCS has been documented (*N* = 45)

**Table 4:** Mortality by systolic blood pressure at arrival

	Systolic blood pressure at arrival ER	
	< 90	≥ 90
Total number of patients	*n* = 69	*n* = 199
Total number of deaths	26	19
Cause of mortality		
Central nervous system	9	10
Exsanguination	5	2
Retroperitoneal exsanguination	6	1
Multi organ failure	2	2
Chest injury	2	1
Cardiac failure	0	1
Respiratory insufficiency	0	1
Pre-existing condition	1	1
Other	1	0

Cause of in-hospital mortality by systolic blood pressure at arrival. Showing systolic blood pressure below 90 has a higher percentage of deaths

Fourteen of the 132 patients (11%) who underwent laparotomy for hemodynamic reasons died due to exsanguination.

### Complications

Unplanned laparotomies occurred in 58 patients (21%) of which 22 were due to relapse of bleeding. In 16 of these patients, rebleeding occurred after adequate resuscitation and return of normal blood pressure was achieved. In six patients, severe coagulopathy was present at this stage, while in ten other patients, diffuse venous (often (retro-hepatic) bleeding occurred despite packing (Table 5).

**Table 5:** Unplanned relaparotomy indications

	Time to index laparotomy (hours)			
	0–2	2–6	6–24	> 24
Total number of patients	190	17	24	37
Total number of relaparotomy	38	5	3	12
Hemodynamic instability^a^	22	0	2	1
- Insufficient source control	6	0	1	1
- Rebleed after resuscitation	10	0	1	0
- Coagulopathy	6	0	0	0
Sepsis				
- Anastomosis leakage	7	1	1	3
- Secondary extension of	3	0	0	3
- contusion over time	2	1	1	0
- Cecum blowout	2	0	0	0
Intra-abdominal abscess	1	1	0	1
Abdominal compartment syndrome	2	0	0	0
Other	10	2	0	4

Indication for unplanned relaparotomy in 58 of all patients after emergency laparotomy. Most of the relaparotomies were performed within 2 h after the index laparotomy or after 24 h. There were several indications for the relaparotomies

^a^Multiple answers possible

Anastomotic leakage occurred in 8.6% (6/69) of all anastomoses. Most of the anastomoses were performed early during the first 2 days after the trauma (55/69); the remaining 14 anastomoses were made during a delayed index laparotomy for a secondary problem (2–4 days after trauma, secondary problems such as ileus, secondary extension of contusion over time, or cecum blow-out). Patients who received an anastomosis in a second procedure following an index laparotomy in damage control surgery were grouped in the first category of early anastomosis. Four patients developed an anastomotic leakage (7.2%) within the early anastomosis group, compared to two patients following an anastomosis after delayed presentation (14.2%).

Furthermore, in 4/94 (4%) cases, an error in judgment regarding the severity of bowel contusion led to intestinal leakage after several days. Incisional hernia occurred in 17 patients (6.3%) and enterocutaneous fistula occurred in one patient (0.4%).

## Discussion

This study demonstrated a steady number of patients who required an emergency laparotomy after trauma performed by a dedicated trauma surgeon in the past decade, with acceptable mortality and complication rates. This study highlights the importance of the preservation of a diverse skillset and experience-based decision making, with dedication to severely injured patients.

A mortality rate of 16.7% was found in the current cohort series, which is comparable to recent literature with mortality rates ranging from 7 to 17% [24–26]. In almost half of our patients, cause of death was considered neurological. Patients who died due to exsanguination were in extremis and suffered severe physiological derangement at presentation with low pH and high base deficit. These findings of severe physiological disturbances are comparable to a large study from Harvin et al., who found an overall mortality rate of 21%, although with slightly different inclusion criteria, since they excluded patients below the age of 18 [2]. A difference between these two case series should be noted, as in the present study most patients died due to neurological injuries, while 65% of deaths was due to hemorrhage by Harvin et al. [2]. This might be a consequence of the differences in numbers of penetrating injuries, which are a frequent indication for laparotomy in the USA or South Africa compared to the Netherlands [27, 28].

Damage control principles were executed in almost 40% of these patients, which require a considerably different decision making strategy compared to an elective orientated process. Trauma surgery often entails easy procedures with complex decision making. The need for a broad skill set and specific expertise is signified by the fact that the patients in this study were severely injured, physiologically deranged, suffered a wide variety of injuries, and required many different intra-abdominal procedures. A dedicated team maintains expertise by ongoing training; for example, the definitive surgical trauma care (DSTC) course [8]. Furthermore, training on the job and protocolized decision making should be institutionalized. Although trauma surgery might be a subspecialty, this specialism however has a wide scope, broad foundation, and therefore with respect to content can be seen as a generalist. Other studies in Europe identified inadequate source control as important cause of (potentially) preventable deaths and assigned this to insufficient expertise [3, 29]. There were 6/132 (4.5%) patients with inadequate source control in the present study. Most of the patients suffering rebleeds were of venous origin after adequate resuscitation demanding the surgeon to stay on top of his/her patient as the resuscitation itself places the patient at risk for deterioration.

Decision making in trauma and critical care is a dynamic process which is accentuated by the patients who required an unplanned relaparotomy. The major reasons for unplanned relaparotomy were bleeding and fecal contamination. Besides damage control laparotomy, resuscitation has a significant role in the treatment of trauma patients with abdominal injuries [30]. After initial damage control surgery with abdominal packing and adequate resuscitation with return of blood volume and pressure, an unplanned laparotomy was prompted mostly due to a diffuse venous rebleed, especially from perihepatic and retroperitoneal origin (11/25). Knowledge of these possible progressions after abdominal trauma is essential for early recognition and treatment [31].

Secondary fecal contamination was found in six patients, either after secondary extension of bowel contusion over time (4/94 judgements; 4%), or after cecum blow-out. Bowel contusion remains a difficult problem both with regard to diagnoses as well as treatment, and highly depends on experience-based decision making [32–34]. An additional six patients developed anastomotic leakage. These results are comparable to literature with anastomotic leakage rates of 2–15% following trauma [35–39]. There was a relatively high number of patients who needed a laparotomy several days after the initial trauma in whom a primary anastomosis was performed (2/14 anastomoses > 48 h; 14.2%). In comparison, 7.2% of the anastomoses developed leakage in patients who underwent their index laparotomy within 2 h after trauma (4/55 anastomoses). This indicates an underestimation of either the bowel condition or the patient’s condition in the delayed group. Patients with delayed laparotomy frequently turned out to have a history of severe alcohol or drugs abuse, which was not apparent during the first days of their admission.

Finally, the non-therapeutic laparotomies could be divided in several groups as well. First, as a result of penetrating injury with disruption of the peritoneum, which in our hospital protocol dictates surgical exploration. Second, severe abdominal pain disproved to be an adequate sole indicator for bowel injury (as this indication resulted in a large number of non-therapeutic laparotomies). Furthermore, non-therapeutic laparotomies were found in conjunction to resuscitation with a resuscitation thoracotomy and either an inconclusive or positive FAST. This can be considered inherent to the patient’s condition and necessity to rule out abdominal blood loss as contributing factor (i.e., during surgery for severe pelvic or chest injury) in the absence of more advanced diagnostic modalities. The role of a pre-operative CT scan can be discussed. When hemodynamically possible, patients will undergo pre-operative CT-scanning. In hemodynamic instability, there is actually no role for CT-scan; even with optimized logistics, this might not be desirable. However, we might have some profit on that behalf when the availability for a CT-scan is easier. Second, our hospital protocol indicates that every penetrating injury in which the fascia is damaged, an exploratory laparotomy must be performed. In half of those patients with negative laparotomy after penetrating trauma, a pre-operative CT-scan was performed.

A similar percentage of patients who required emergency laparotomy after trauma was found compared to other European studies from level 1 trauma centers in the past decades [3, 40]. It is therefore likely that this percentage will remain steady in the near future. Although 10 years ago concerns were raised to maintain sufficient expertise in these settings, the residency program and ongoing training programs demonstrate similar or even somewhat improved outcomes in the current study [41]. Furthermore, dedication to trauma both with regard to physicians and logistics might have contributed to these results [17]. A trauma center should be prepared for the severely injured trauma patient that requires an emergency laparotomy. A rapid and adequate response to hemodynamically unstable patients is required to lower mortality rates [42, 43]. In our hospital, it is the trauma surgeon with a background in general surgery who is leading the trauma team and performs all truncal and musculoskeletal (damage control) operative procedures. Nearly all patients arrived in the operating room within a reasonable short timeframe and the average operation time was 62 min. It should be noted that in our hospital, no CT scan is available in the crash room (which leads to transfer times), and there is no trauma dedicated hybrid operating room [44–50]. These factors did have major impact on the decision making represented in the present study, but might be considered difficult to extract in a retrospective design. However, despite the limitations of a retrospective study design, essential information was over 90% complete, which makes the outcome data rather robust. Thus, optimizing logistics for both surgical procedures and resuscitation might further improve mortality and functional outcomes.

## Conclusion

Regardless of all the advances in the non-operative management of blunt abdominal trauma patients, a persistent number of injured patients require an emergency laparotomy. A dedicated surgical team with experience and maintenance in damage control surgery can achieve acceptable outcomes in terms of mortality and morbidity rates, although further centralization of these patients might be warranted to further optimize logistics and efficiency.

## Data Availability

The datasets used and/or analyzed during the current study are available from the corresponding author on reasonable request.

## Abbreviations

NOM: Non-operative management
DCS: Damage control surgery
UMCU: University Medical Center Utrecht
ICU: Intensive care unit
OR: Operation room
ATLS: Advanced trauma life support
ASA: American Society of Anesthesiologists
ISS: Injury Severity Score
GCS: Glasgow Coma Scale
IQR: Interquartile range
IRB: Institutional review board
DSTC: Definitive surgical trauma care

## References

[1] Groven S, Gaarder C, Eken T, Skaga NO, Naess PA (2014). Abdominal injuries in a major Scandinavian trauma center—performance assessment over an 8 year period. J Trauma Manag Outcomes.

[2] Harvin JA, Maxim T, Inaba K, Martinez-Aguilar MA, King DR, Choudhry AJ (2017). Mortality after emergent trauma laparotomy: a multicenter, retrospective study. J Trauma Acute Care Surg.

[3] van Gool MH, Giannakopoulos GF, Geeraedts LM, de Lange-de KES, Zuidema WP (2015). Complications after laparotomy for trauma: a retrospective analysis in a level I trauma centre. Langenbecks Arch Surg.

[4] Markogiannakis H, Sanidas E, Messaris E, Michalakis I, Kasotakis G, Melissas J (2006). Management of blunt hepatic and splenic trauma in a Greek level I trauma centre. Acta Chir Belg.

[5] Ciesla DJ, Moore EE, Cothren CC, Johnson JL, Burch JM (2006). Has the trauma surgeon become house staff for the surgical subspecialist?. Am J Surg.

[6] Chowdhury S, Navsaria PH, Edu S, Nicol AJ (2016). The effect of emergency medical services response on outcome of trauma laparotomy at a Level 1 Trauma Centre in South Africa. S Afr J Surg.

[7] Pekkari P, Bylund PO, Lindgren H, Oman M (2014). Abdominal injuries in a low trauma volume hospital—a descriptive study from northern Sweden. Scand J Trauma Resusc Emerg Med.

[8] Jacobs LM, Lorenzo C, Brautigam RT (2001). Definitive surgical trauma care live porcine session: a technique for training in trauma surgery. Conn Med.

[9] Undurraga Perl VJ, Leroux B, Cook MR, Watson J, Fair K, Martin DT (2016). Damage-control resuscitation and emergency laparotomy: Findings from the PROPPR study. J Trauma Acute Care Surg.

[10] Hietbrink F, Simmermacher RKJ, de Vries MB, van Wessem KJP, de Jong MB, Leenen LPH (2017). Emergency laparotomy in a trauma patient. Ned Tijdschr Geneeskd.

[11] Bergeron E, Lavoie A, Belcaid A, Moore L, Clas D, Razek T (2007). Surgical management of blunt thoracic and abdominal injuries in Quebec: a limited volume. J Trauma.

[12] Watson JJ, Nielsen J, Hart K, Srikanth P, Yonge JD, Connelly CR (2017). Damage control laparotomy utilization rates are highly variable among Level I trauma centers: pragmatic, randomized optimal platelet and plasma ratios findings. J Trauma Acute Care Surg.

[13] Germanos S, Gourgiotis S, Villias C, Bertucci M, Dimopoulos N, Salemis N (2008). Damage control surgery in the abdomen: an approach for the management of severe injured patients. Int J Surg.

[14] Stone HH, Strom PR, Mullins RJ (1983). Management of the major coagulopathy with onset during laparotomy. Ann Surg.

[15] Rotondo MF, Schwab CW, McGonigal MD, Phillips GR, Fruchterman TM, Kauder DR (1993). ‘Damage control’: an approach for improved survival in exsanguinating penetrating abdominal injury. J Trauma.

[16] Oshun N, Hardcastle TC (2015). Validation of the mortality prediction equation for damage control surgery in an independent severe trauma population. S Afr J Surg.

[17] van der Vliet QMJ, van Maarseveen OEC, Smeeing DPJ, Houwert RM, van Wessem KJP, Simmermacher RKJ, et al. Severely injured patients benefit from in-house attending trauma surgeons. Injury 2018. 10.1016/j.injury.2018.08.006 30119939

[18] Benz D, Balogh ZJ (2017). Damage control surgery: current state and future directions. Curr Opin Crit Care.

[19] Weber DG, Bendinelli C, Balogh ZJ (2014). Damage control surgery for abdominal emergencies. Br J Surg.

[20] Goslings JC, Ponsen KJ, Luitse JS, Jurkovich GJ (2006). Trauma surgery in the era of nonoperative management: the Dutch model. J Trauma.

[21] Waibel BH, Rotondo MM (2012). Damage control surgery: it’s evolution over the last 20 years. Rev Col Bras Cir.

[22] van Wessem KJ (2010). Possible devices to temporary cover the open abdomen: pros and cons. Acta Chir Belg.

[23] Timmers TK, Verhofstad MH, Leenen LP (2015). Intensive care organisation: Should there be a separate intensive care unit for critically injured patients?. World J Crit Care Med.

[24] Bowie JM, Badiee J, Calvo RY, Sise MJ, Wessels LE, Butler WJ (2019). Outcomes after single-look trauma laparotomy: A large population-based study. J Trauma Acute Care Surg.

[25] Hasler RM, Nuesch E, Juni P, Bouamra O, Exadaktylos AK, Lecky F (2011). Systolic blood pressure below 110 mm Hg is associated with increased mortality in blunt major trauma patients: multicentre cohort study. Resuscitation.

[26] Joseph B, Azim A, Zangbar B, Bauman Z, O'Keeffe T, Ibraheem K (2017). Improving mortality in trauma laparotomy through the evolution of damage control resuscitation: Analysis of 1,030 consecutive trauma laparotomies. J Trauma Acute Care Surg.

[27] Lansink KW, Gunning AC, Leenen LP (2013). Cause of death and time of death distribution of trauma patients in a Level I trauma centre in the Netherlands. Eur J Trauma Emerg Surg.

[28] Matsushima K, Khor D, Berona K, Antoku D, Dollbaum R, Khan M (2018). Double jeopardy in penetrating trauma: Get FAST, Get It Right. World J Surg.

[29] Girard E, Jegousso Q, Boussat B, Francois P, Ageron FX, Letoublon C (2019). Preventable deaths in a French regional trauma system: a six-year analysis of severe trauma mortality. J Visc Surg.

[30] Duchesne JC, Kimonis K, Marr AB, Rennie KV, Wahl G, Wells JE (2010). Damage control resuscitation in combination with damage control laparotomy: a survival advantage. J Trauma.

[31] Lin BC, Fang JF, Chen RJ, Wong YC, Hsu YP (2014). Surgical management and outcome of blunt major liver injuries: experience of damage control laparotomy with perihepatic packing in one trauma centre. Injury.

[32] Cripps NP, Cooper GJ (1997). Risk of late perforation in intestinal contusions caused by explosive blast. Br J Surg.

[33] Eshraghi N, Mullins RJ, Mayberry JC, Brand DM, Crass RA, Trunkey DD (1998). Surveyed opinion of American trauma surgeons in management of colon injuries. J Trauma.

[34] Geyer LL, Korner M, Linsenmaier U, Huber-Wagner S, Kanz KG, Reiser MF (2013). Incidence of delayed and missed diagnoses in whole-body multidetector CT in patients with multiple injuries after trauma. Acta Radiol.

[35] Demetriades D (2004). Colon injuries: new perspectives. Injury.

[36] Dente CJ, Patel A, Feliciano DV, Rozycki GS, Wyrzykowski AD, Nicholas JM (2005). Suture line failure in intra-abdominal colonic trauma: is there an effect of segmental variations in blood supply on outcome?. J Trauma.

[37] Oosthuizen GV, Kong VY, Estherhuizen T, Bruce JL, Laing GL, Odendaal JJ (2018). The impact of mechanism on the management and outcome of penetrating colonic trauma. Ann R Coll Surg Engl.

[38] Schnuriger B, Inaba K, Wu T, Eberle BM, Belzberg H, Demetriades D (2011). Crystalloids after primary colon resection and anastomosis at initial trauma laparotomy: excessive volumes are associated with anastomotic leakage. J Trauma.

[39] Sharpe JP, Magnotti LJ, Weinberg JA, Parks NA, Maish GO, Shahan CP (2012). Adherence to a simplified management algorithm reduces morbidity and mortality after penetrating colon injuries: a 15-year experience. J Am Coll Surg.

[40] Gaski IA, Skattum J, Brooks A, Koyama T, Eken T, Naess PA (2018). Decreased mortality, laparotomy, and embolization rates for liver injuries during a 13-year period in a major Scandinavian trauma center. Trauma Surg Acute Care Open.

[41] Gaarder C, Skaga NO, Eken T, Pillgram-Larsen J, Buanes T, Naess PA (2005). The impact of patient volume on surgical trauma training in a Scandinavian trauma centre. Injury.

[42] Barbosa RR, Rowell SE, Fox EE, Holcomb JB, Bulger EM, Phelan HA (2013). Increasing time to operation is associated with decreased survival in patients with a positive FAST examination requiring emergent laparotomy. J Trauma Acute Care Surg.

[43] Schechtman D, He JC, Zosa BM, Allen D, Claridge JA (2017). Trauma system regionalization improves mortality in patients requiring trauma laparotomy. J Trauma Acute Care Surg.

[44] Gross T, Messmer P, Amsler F, Fuglistaler-Montali I, Zurcher M, Hugli RW (2010). Impact of a multifunctional image-guided therapy suite on emergency multiple trauma care. Br J Surg.

[45] Sierink JC, Treskes K, Edwards MJ, Beuker BJ, den HD, Hohmann J (2016). Immediate total-body CT scanning versus conventional imaging and selective CT scanning in patients with severe trauma (REACT-2): a randomised controlled trial. Lancet.

[46] Peev MP, Chang Y, King DR, Yeh DD, Kaafarani H, Fagenholz PJ (2015). Delayed laparotomy after selective non-operative management of penetrating abdominal injuries. World J Surg.

[47] Fikry K, Velmahos GC, Bramos A, Janjua S, de MM KDR (2011). Successful selective nonoperative management of abdominal gunshot wounds despite low penetrating trauma volumes. Arch Surg.

[48] Johnson JJ, Garwe T, Raines AR, Thurman JB, Carter S, Bender JS (2013). The use of laparoscopy in the diagnosis and treatment of blunt and penetrating abdominal injuries: 10-year experience at a level 1 trauma center. Am J Surg.

[49] Leenen LP (2009). Abdominal trauma: from operative to nonoperative management. Injury.

[50] Malinoski DJ, Patel MS, Yakar DO, Green D, Qureshi F, Inaba K (2010). A diagnostic delay of 5 hours increases the risk of death after blunt hollow viscus injury. J Trauma.

